# Defining the Normal Growth Curve of Fetal Fractional Limb Volume in a Japanese Population

**DOI:** 10.3390/jcm10030485

**Published:** 2021-01-29

**Authors:** Satoru Ikenoue, Yohei Akiba, Toyohide Endo, Yoshifumi Kasuga, Kazumi Yakubo, Ryota Ishii, Mamoru Tanaka, Daigo Ochiai

**Affiliations:** 1 Department of Obstetrics and Gynecology, Keio University School of Medicine, Tokyo 160-8582, Japan; e2toyo26@gmail.com (T.E.); 17yoshi23.k@gmail.com (Y.K.); mtanaka@keio.jp (M.T.); ochiaidaigo@gmail.com (D.O.); 2 Department of Obstetrics and Gynecology, Saitama Municipal Hospital, Saitama 336-8522, Japan; m30002youhei@gmail.com (Y.A.); k-yakubo@tiara.ocn.ne.jp (K.Y.); 3 Biostatistics Unit, Clinical and Translational Research Center, Keio University Hospital, Tokyo 160-8582, Japan; rishii@keio.jp

**Keywords:** fetal soft tissue, fetal ultrasound, fractional arm volume, fractional limb volume, fractional thigh volume, Japanese population, third trimester of gestation

## Abstract

Fetal fractional limb volume is a useful measure for predicting birth weight and newborn adiposity; however, a normal growth curve has been reported solely in the United States. As the birth weight of neonates in Japan is significantly lower than that in the US, fetal fractional limb volume is likely to be smaller in the Japanese population. This study aimed to define the normal growth curve of fractional arm volume (AVol) and thigh volume (TVol) in the Japanese population. Ultrasound scans of 453 AVol and TVol pairs were obtained; each AVol and TVol percentile at each gestational age was calculated. The measured AVol and TVol at each gestational week were also converted to z-scores based on a previous report. The growth curves increased linearly until the second trimester and exponentially in the third trimester. Linear regression showed a significant negative correlation between gestational age and AVol and TVol z-scores. The growth pattern of fetal fractional limb volume in the Japanese population is consistent with, but smaller than, that reported in the US; this difference becomes greater as the gestational age progresses.

## 1. Introduction

Intrauterine fetal growth is an important parameter for predicting perinatal outcomes [1,2], as well as metabolic compromise of the offspring in later life [3]. Conventionally, estimated fetal weight is mainly derived from composite skeletal measurements (biparietal diameter and femur diaphysis length) without regard to soft tissue development of the fetus [4]. Although abdominal circumference is one of the components used to estimate fetal weight and includes soft tissue (circumferential subcutaneous fat), it is largely affected by fetal liver size [4]. Additionally, birth weight estimation is known to generate errors as great as 15% from actual birth weight [5,6], especially when estimating the fetal weight of small- or large-for-gestational-age fetuses [7]. This is potentially because conventional fetal weight estimation does not include fetal soft tissue (muscle and fat deposition) parameters, and small- or large-for-gestational-age fetuses have a greater variation in soft tissue volume [8].

Fetal fractional limb volume has been proposed as a useful measure for quantifying fetal soft tissue volume, and it has proven to be useful for improving birth weight estimation [9,10]. Fractional limb volume is composed of both fat and lean mass, accounting for a higher proportion of variation in newborn adiposity (body fat percentage) than conventional fetal measures (e.g., estimated fetal weight) [11]. Hence, fractional limb volume has the potential to distinguish malnourished or over-nourished fetuses from fetuses that are constitutionally small or large, but otherwise normal.

The reference range of fetal fractional limb volume at each gestational age has been previously reported by Lee et al. for the United States (among a cohort comprising 58% Caucasian, 32% African-American, 6% Asian, and 4% Hispanic or other races) [12]. It is well established that the birth weight and anthropomorphic measures of Japanese neonates are significantly lower than those of neonates in Europe and the United States [13]; hence, it is suspected that fetal fractional limb volume would be smaller in the Japanese population. To the best of our knowledge, this has not been systematically investigated and reported thus far in Japan. Clarifying and recognizing the characteristics of fractional limb volume growth in the Japanese population can lead to the development of an accurate reference model of birth weight and newborn adiposity in Japanese neonates.

Therefore, the aims of the study were as follows: (1) to define the normal growth curve of fetal fractional limb volume across gestation in the Japanese population, and (2) to compare the difference in the growth curves of fractional limb volume between the Japanese and the United States populations. Considering that the fractional limb volume includes fetal soft tissue (i.e., fat and muscle), and fetal soft tissue growth accelerates in the early third trimester [8,14], we hypothesized that the fractional limb volume in the Japanese population would be smaller than that reported in a previous study, and the difference would become greater as gestational age proceeds, particularly in the third trimester of gestation.

## 2. Materials and Methods

### 2.1. Study Population

The current study was an exploratory observational study in which Japanese women with singleton pregnancies were prospectively enrolled between July 2017 and June 2020. The study was approved by Saitama Municipal Hospital Ethical Committee (No. A-3013), and informed consent was obtained from the mothers. Exclusion criteria included multiple gestations, uterine anomalies, chronic hypertension, pregestational diabetes, smoking, congenital malformations, and chromosomal abnormalities.

### 2.2. Prenatal Ultrasonography

Fetal fractional limb volume was assessed between 20 and 40 weeks’ gestation. Fractional arm volume (AVol) and thigh volume (TVol) were measured as cylindrical limb volumes based on 50% of the total diaphysis length (to eliminate the proximal or distal end of the diaphysis where the soft tissue boundaries are poorly visualized), using 4D View software (GE Healthcare, Milwaukee, WI, USA), as previously reported [6]. The acquired partial limb volume was divided into five subsections of equal length, and the contour of each subsection was traced manually in the axial view. Each of the measurements was obtained in duplicate and averaged [15].

All ultrasound scans were obtained by using the Voluson E10 (GE Healthcare) with a matrix array transducer (RM6C) by one of the two obstetricians with training in fetal ultrasonography (S.I. and Y.A.). The reproducibility analyses were conducted with 40 AVol and TVol measurements performed by each ultrasonographer. The interobserver coefficients of variation between the two ultrasonographers were 5.8% for AVol and 6.3% for TVol, while the intraobserver coefficients of variation of each ultrasonographer were 4.5% for AVol and 4.9% for TVol [15]. Bland–Altman plots are also provided in Supplementary Materials Figure S1. The AVol and TVol at each gestational week were converted to z-scores based on a previous report of the normal reference range of fractional volume in the United States population [12].

### 2.3. Birth Outcomes

Birth weight and infant sex were abstracted from the medical records. Birth weight was normalized by using the Japanese birth weight percentile curves standardized for gestational age at birth and for sex [16]. Gestational age was calculated based on the last menstrual period and confirmed via ultrasonography in the first trimester of gestation, as per standard clinical criteria [17]. If the difference between the menstrual date and the ultrasound date based on the crown rump length [18] was greater than 5 days, the estimated due date was calculated by the ultrasound gestational age assessment [17,19].

### 2.4. Pre-Pregnancy Body Mass Index and Gestational Weight Gain

Pre-pregnancy weight obtained by a maternal self-report and height measured at the first antenatal visit were used to calculate pre-pregnancy body mass index (BMI). Gestational weight gain was calculated as the pre-pregnancy weight subtracted from the weight clinically recorded within 1 week before delivery.

### 2.5. Statistical Analysis

The scatterplots of fractional limb volume measurements and gestational age indicated a curvilinear relationship, with increasing variability (heteroscedasticity across gestational ages). Box-Cox transformations for AVol and TVol were used to achieve the most appropriate linear relationship between the transformed volume and gestational age. After the transformation, we applied the linear mixed-effect model to account for repeated measurements within the subject. The linear mixed-effect model was defined as the following form:

Transformed volume = (β_0_ + *b*_i_) × β_i_ × gestational age, (1)

where β_0_ and β_i_ are the fixed effect parameters and b_i_ is the random effect parameter for the ith subject. Based on these results, 5th, 10th, 25th, 50th, 75th, 90th, and 95th percentiles in the transformed scale were calculated, and these percentiles were then transformed back into the original scale. Linear regression analyses were also performed to determine the association between the z-score of the fractional limb volume and gestational age at the time of the ultrasound scan. All statistical analyses were performed by using SAS, version 9.4 (SAS Institute, Cary, NC, USA). Two-sided *p*-values less than 0.05 were considered statistically significant.

## 3. Results

We included 247 Japanese women with singleton pregnancy; maternal and neonatal characteristics are shown in Table 1. Overall, 453 ultrasound scans were obtained, with a mean of 1.8 per pregnancy. The number of scans at each gestational age and the calculated percentile ranges for AVol and TVol are summarized in Table 2.

**Figure 1:**
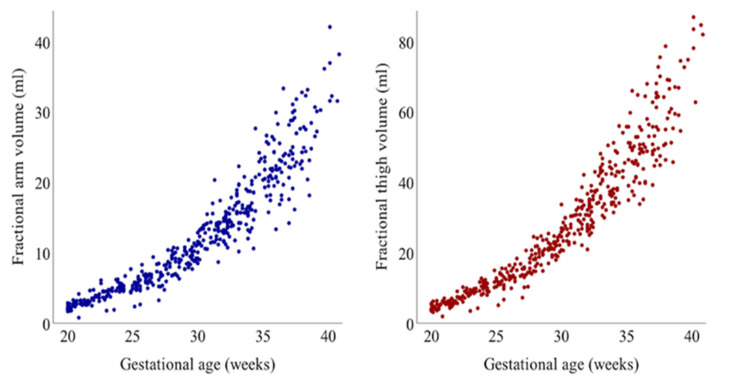
Scatterplot of fractional arm volume and thigh volume across gestation.

**Figure 2:**
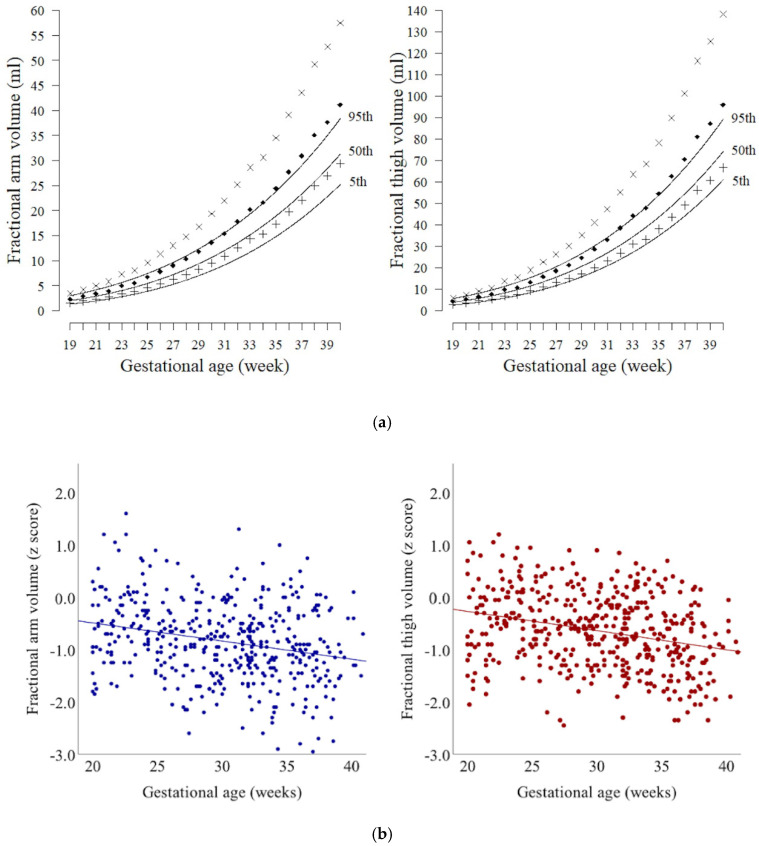
(**a**) Growth curve of fractional arm volume and thigh volume in the present cohort (continuous lines) and that of previous report [12] (interrupted lines; ×, 95th; ◆, 50th; +, 5th percentile). (**b**) The z-scores of arm volume (AVol) and thigh volume (TVol) across gestation, with the linear regression lines, which are negatively significant (*p* < 0.0001).

The scatterplots of AVol and TVol across gestation are shown in Figure 1, while the growth trajectories of AVol and TVol are depicted in Figure 2a. The fractional limb volume of the present cohort increased linearly with respect to gestational age, until the second trimester of gestation; it then increased exponentially in the third trimester, which is compatible with a previous report [12]. The previously reported 50th percentile curves of AVol and TVol in the United States population were above the 95th percentile curve of those in the present Japanese cohort. The scatterplots and regression lines of the association between gestational age and z-score of AVol and TVol are shown in Figure 2b. The average z-scores of both AVol and Tvol were below zero across gestational age, while the linear regression lines showed a significant negative correlation (*p* < 0.0001).

## 4. Discussion

### 4.1. Principal Findings

The present study is the first to define the normal growth trajectory of fetal fractional limb volume in the Japanese population. The fractional limb volume increased linearly until the second trimester of gestation; it then increased exponentially in the third trimester. The fractional limb volume of the Japanese fetus was smaller than reported for neonates in the United States [12].

### 4.2. Clinical Implications

The fractional limb volume growth curve indicated a linear increase with respect to gestational age until the second trimester of gestation; it then increased exponentially in the third trimester. This growth pattern replicated the prior report of fractional limb volume growth in the United States [12]. Fractional limb volume is composed of fat mass and lean mass; the critical time of fractional limb volume growth acceleration is in the early third trimester [20,21], which coincides with the period of accelerated fetal fat deposition [8,14]. Fetal fat deposition is histologically evident at 14–16 weeks of gestation [22]; however, fetal soft tissue has been reported to accumulate mainly in later gestation, while fat deposition typically accelerates after 30 weeks of gestation [8,14]. Human infants have a substantially higher percentage of body fat, as well as larger brains than other mammals [23]. Adipose tissue plays an important role in human infants because it constitutes a key buffer against the limited nutrient supply, particularly to meet the brain’s energy requirements during early postnatal life [23,24]. Adipose-tissue-derived ketone bodies can provide as much as 25% of the energy required by the brain [25]. During pregnancy, fat deposition is estimated to represent over half of the calorie accretion from 27 weeks of gestation until term, constituting up to 90% of this metabolic requirement before delivery [26]. This evidence would explain the exponential increase of the fractional limb volume in the third trimester of gestation.

The growth trajectories of AVol and TVol in the present cohort were compared with the results of the previous study conducted in the United States [12]. The 50th percentile curves of AVol and TVol in the United States population were above the 95th percentile curve of those in the present cohort. This comparison lacks a statistical assessment; however, it is apparent that a difference in the fractional limb volume exists between the two cohorts, particularly in the third trimester of gestation. Fractional limb volume measurements at each gestational week in the present population were also converted to z-scores, based on the previous report [12], and the average z-scores of both AVol and Tvol in the Japanese population were below zero across gestation. Moreover, the linear regression lines showed a significant negative correlation between gestational age and the z-scores of AVol and TVol. These findings indicate that AVol and TVol in the Japanese population are smaller than those of neonates in the United States population across gestation, and the difference is greater as the gestational age progresses. Defining the normal growth curve of fractional limb volume in the Japanese population, which has been demonstrated in the present cohort, is essential to expand the use of fractional limb volume to better predict fetal growth and birth weight in the Japanese population.

### 4.3. Research Implications

Measuring fractional limb volume is significant, as it is a quantitative soft tissue assessment of the limb that may provide a useful surrogate marker of fetal nutritional status [4,15,27]. This can distinguish malnourished or over-nourished fetuses from fetuses that are constitutionally small or large. Serial fetal growth data, including fractional limb volume and related measurements, could be applied to the “individualized growth assessment” tool in the Japanese population. Individualized growth assessment can estimate and generate individual fetal growth and birth weight trajectories in the third trimester of gestation, based on the growth velocity of fetal size parameters, including fractional limb volume in the second trimester [21,28]. Defining the normal growth curve of fractional limb volume in the Japanese population is useful as this individualized growth assessment tool can be applied to the Japanese population. The ability to detect and recognize variations in fetal fractional limb volume in the Japanese population may provide a greater insight into understanding the origins of pathological alterations of fetal growth in small- or large-for-gestational-age fetuses in the Japanese population. The preliminary analysis of our data suggests that fractional limb volume in the Japanese population appears smaller than that reported in the United States population, especially in the third trimester of gestation. Future prospective longitudinal studies, including different races and ethnicities in a larger cohort, will extend this preliminary observation in a comparative study with respect to fractional limb volume. This would be an important contribution to the field of fetal growth and body composition assessment.

AVol and TVol are indicators of soft tissue, including both fat mass and lean mass. Further consideration of other fetal measures that can better differentiate between these parameters (e.g., fractional limb fat volume or lean volume) may highlight a more precise marker of altered fetal growth and body composition, and this also warrants further investigation.

### 4.4. Strength and Limitations

The strengths of our study include the measures of fetal fractional limb volume in a well-characterized cohort in a homogenous Japanese population. The participants in this study were of maternal age and had pregravid BMIs and gestational weight gains that were standard for the Japanese population. A possible limitation is that the difference in the growth trajectories of fractional limb volume between the Japanese population and the United States population was not statistically examined because the actual values of the fractional limb volume measured at each gestational age and the maternal demographic characteristics in the United States population were not available. Maternal age, parity, pregravid BMI, and gestational weight gain are well-established factors that affect birth weight and newborn adiposity [29,30], and these factors have a potentially significant effect on the growth of fractional limb volume. The Japanese population is known to have a significantly lower pregravid BMI than the United States population. The present study did not fully consider these factors that could statistically affect the growth of fetal fractional volume. Further study is warranted to investigate whether fetal fractional limb volume measured in the Japanese population is related with accuracy of fetal weight estimation. Moreover, a multi-institutional longitudinal study is required to replicate the present study, to confirm the growth curve of the fractional limb volume with a more representative Japanese population. The number of observations at the last two weeks of gestation is small, which might affect the normal range of fractional limb volume at 39 and 40 weeks’ gestation.

## 5. Conclusions

The present study defined the normal growth curve of fetal fractional limb volume in the Japanese population across gestation. The growth pattern of fractional limb volume in the Japanese population is compatible with the results of the United States population; however, there was a larger difference in the third trimester of gestation. The present findings could help develop an accurate reference model to predict birth weight and newborn adiposity in the Japanese population.

## Figures and Tables

**Table 1:** Maternal and newborn demographic and clinical characteristics (*n* = 247).

Characteristics	*n* (%) or Mean ± SD
**Maternal Characteristics**	
Age, years	32.5 ± 5.7
Pre-pregnancy BMI, kg/m^2^	21.7 ± 3.2
Underweight (BMI < 18.5)	27 (11%)
Normal weight (18.5 ≤ BMI < 25)	188 (76%)
Overweight/Obese (BMI ≥ 25.0)	32 (13%)
Gestational weight gain, kg	9.1 ± 4.5
Primiparous	128 (52%)
**Newborn Characteristics**	
Gestational age at birth, weeks	38.0 ± 2.3
Birth weight, g	2841 ± 503
Birth weight percentile, %	53.2 ± 27.5
Small for gestational age (birth weight < 10th centile)	19 (8%)
Appropriate for gestational age	205 (83%)
Large for gestational age (birth weight ≥ 90th centile)	23 (9%)
Infant sex (female)	122 (49%)

**Table 2:** Fitted percentiles of fractional limb volume.

Gestational Age (Weeks)	*n*	Fractional Arm Volume							Fractional Thigh Volume						
		5th	10th	25th	50th	75th	90th	95th	5th	10th	25th	50th	75th	90th	95th
20	23	1.7	1.8	2.0	2.4	2.8	3.1	3.3	3.4	3.6	4.0	4.6	5.2	5.9	6.2
21	20	2.0	2.1	2.4	2.8	3.3	3.7	3.9	4.2	4.4	4.9	5.6	6.3	7.1	7.5
22	17	2.5	2.6	2.9	3.4	3.8	4.3	4.5	5.1	5.4	6.0	6.8	7.6	8.4	8.8
23	16	2.9	3.0	3.4	3.9	4.5	5.0	5.3	6.2	6.5	7.2	8.1	9.1	10.0	10.5
24	20	3.4	3.6	4.0	4.6	5.2	5.8	6.1	7.4	7.8	8.6	9.6	10.7	11.8	12.4
25	22	4.0	4.2	4.7	5.4	6.1	6.7	7.0	8.8	9.3	10.2	11.3	12.6	13.8	14.5
26	18	4.7	4.9	5.5	6.2	7.0	7.7	8.1	10.4	10.9	12.0	13.3	14.7	16.0	16.8
27	22	5.4	5.7	6.3	7.1	8.0	8.8	9.2	12.2	12.8	14.0	15.5	17.0	18.5	19.4
28	22	6.3	6.6	7.3	8.2	9.1	10.0	10.5	14.2	14.9	16.3	17.9	19.6	21.3	22.4
29	29	7.1	7.5	8.3	9.3	10.3	11.4	12.0	16.4	17.3	18.8	20.6	22.5	24.4	25.6
30	23	8.2	8.6	9.5	10.6	11.7	12.8	13.4	19.0	20.0	21.7	23.6	25.8	27.8	29.2
31	24	9.3	9.8	10.8	11.9	13.2	14.4	15.1	21.8	22.9	24.8	27.0	29.3	31.5	33.1
32	37	10.5	11.1	12.2	13.5	14.8	16.1	16.9	24.9	26.2	28.2	30.6	33.2	35.7	37.5
33	31	11.9	12.5	13.7	15.1	16.6	18.0	18.9	28.3	29.8	32.0	34.7	37.5	40.2	42.2
34	23	13.4	14.1	15.4	16.9	18.5	20.1	21.1	32.0	33.7	36.2	39.1	42.2	45.1	47.4
35	26	15.0	15.8	17.2	18.9	20.6	22.3	23.4	36.2	38.1	40.8	44.0	47.3	50.5	53.0
36	24	16.8	17.7	19.2	21.0	22.9	24.7	25.9	40.7	42.8	45.8	49.2	52.9	56.3	59.1
37	26	18.7	19.7	21.3	23.3	25.3	27.3	28.7	45.6	48.0	51.2	55.0	58.9	62.7	65.8
38	17	20.8	21.9	23.7	25.7	28.0	30.1	31.6	50.9	53.6	57.1	61.2	65.5	69.5	73.0
39	7	23.1	24.3	26.2	28.4	30.8	33.1	34.8	56.7	59.7	63.5	67.9	72.6	76.9	80.7
40	6	25.5	26.8	28.9	31.3	33.8	36.3	38.1	63.0	66.3	70.4	75.2	80.2	84.9	89.1

## Data Availability

The data presented in this study are available on reasonable request from the corresponding author.

## Supplementary Materials

The following are available online at https://www.mdpi.com/2077-0383/10/3/485/s1, Figure S1: Reproducibility of fractional limb volume measurements. Bland–Altman plots indicate an interobserver bias and agreement for fractional arm volume (AVol) and thigh volume (TVol) measurements. Horizontal lines are the average measurement bias in terms of percentage differences and the 95% limits of agreement for these percentage differences.

## Abbreviations

BMI	body mass index
AVol	fractional arm volume
TVol	fractional thigh volume
