# Supplementary figures and images for: Defining the Normal Growth Curve of Fetal Fractional Limb Volume in a Japanese Population
Source: J Clin Med. 2021 Jan 29;10(3):485. doi: 10.3390/jcm10030485 (PMC7866269; doi:10.3390/jcm10030485)

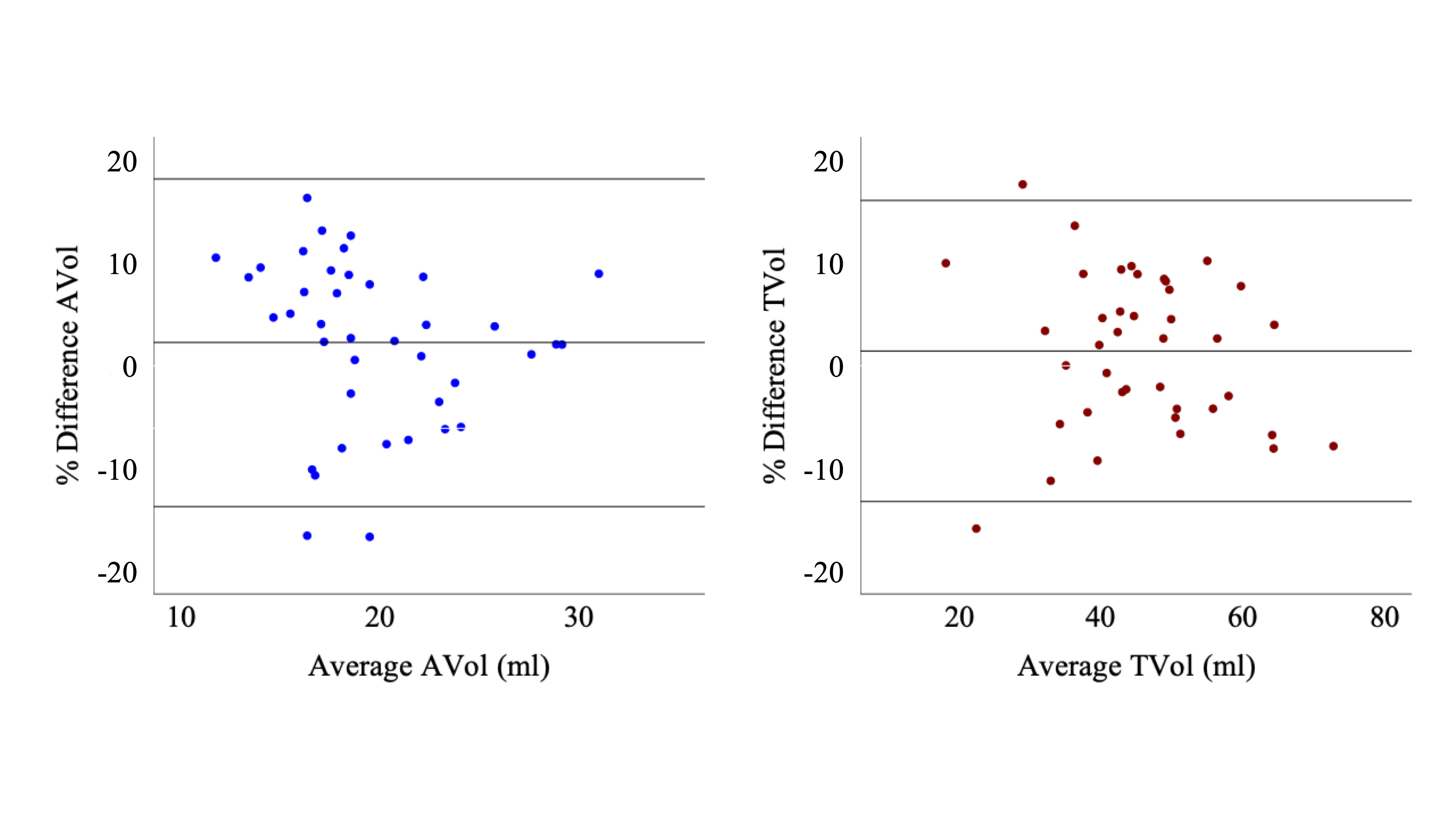

Supplement: Supplementary file 1 [file jcm-10-00485-s001.zip › jcm-1088857-supplementary.tiff]
